# Potential Links between Hepadnavirus and Bornavirus Sequences in the Host Genome and Cancer

**DOI:** 10.3389/fmicb.2017.02537

**Published:** 2017-12-19

**Authors:** Tomoyuki Honda

**Affiliations:** Division of Virology, Department of Microbiology and Immunology, Osaka University Graduate School of Medicine, Osaka, Japan

**Keywords:** hepatitis B virus, endogenous viral elements, cancer, borna disease virus, non-coding RNAs, LINE-1, retrotransposon

## Abstract

Various viruses leave their sequences in the host genomes during infection. Such events occur mainly in retrovirus infection but also sometimes in DNA and non-retroviral RNA virus infections. If viral sequences are integrated into the genomes of germ line cells, the sequences can become inherited as endogenous viral elements (EVEs). The integration events of viral sequences may have oncogenic potential. Because proviral integrations of some retroviruses and/or reactivation of endogenous retroviruses are closely linked to cancers, viral insertions related to non-retroviral viruses also possibly contribute to cancer development. This article focuses on genomic viral sequences derived from two non-retroviral viruses, whose endogenization is already reported, and discusses their possible contributions to cancer. Viral insertions of hepatitis B virus play roles in the development of hepatocellular carcinoma. Endogenous bornavirus-like elements, the only non-retroviral RNA virus-related EVEs found in the human genome, may also be involved in cancer formation. In addition, the possible contribution of the interactions between viruses and retrotransposons, which seem to be a major driving force for generating EVEs related to non-retroviral RNA viruses, to cancers will be discussed. Future studies regarding the possible links described here may open a new avenue for the development of novel therapeutics for tumor virus-related cancers and/or provide novel insights into EVE functions.

## Introduction

Viruses can deposit their sequences into the host genome during infection. Consistently, animal genomes contain many viral-related sequences, called endogenous viral elements (EVEs) (Katzourakis and Gifford, 2010; Holmes, 2011; Parrish and Tomonaga, 2016). EVEs are mainly derived from ancient retroviruses because retroviruses require the integration of their DNAs into the host genome for replication. In addition to retroviruses, DNA and non-retroviral RNA viruses can sometimes become integrated into the host genome, despite the fact that integration events are not required for the viral life cycle. In particular, sequences of non-retroviral RNA viruses seem to have been integrated into the host genome possibly by machineries of a host retrotransposon, long interspersed nuclear element 1 (LINE-1, or L1) (Horie et al., 2010). The integration events of viral sequences occur not only in somatic cells but also in germ line cells. If the integration event occurs in germ line cells, the integrated viral sequences become inherited as EVEs. Thus, the integration of viral sequences into the genome of germ line cells is an essential first step for generating EVEs.

**FIGURE 1:**
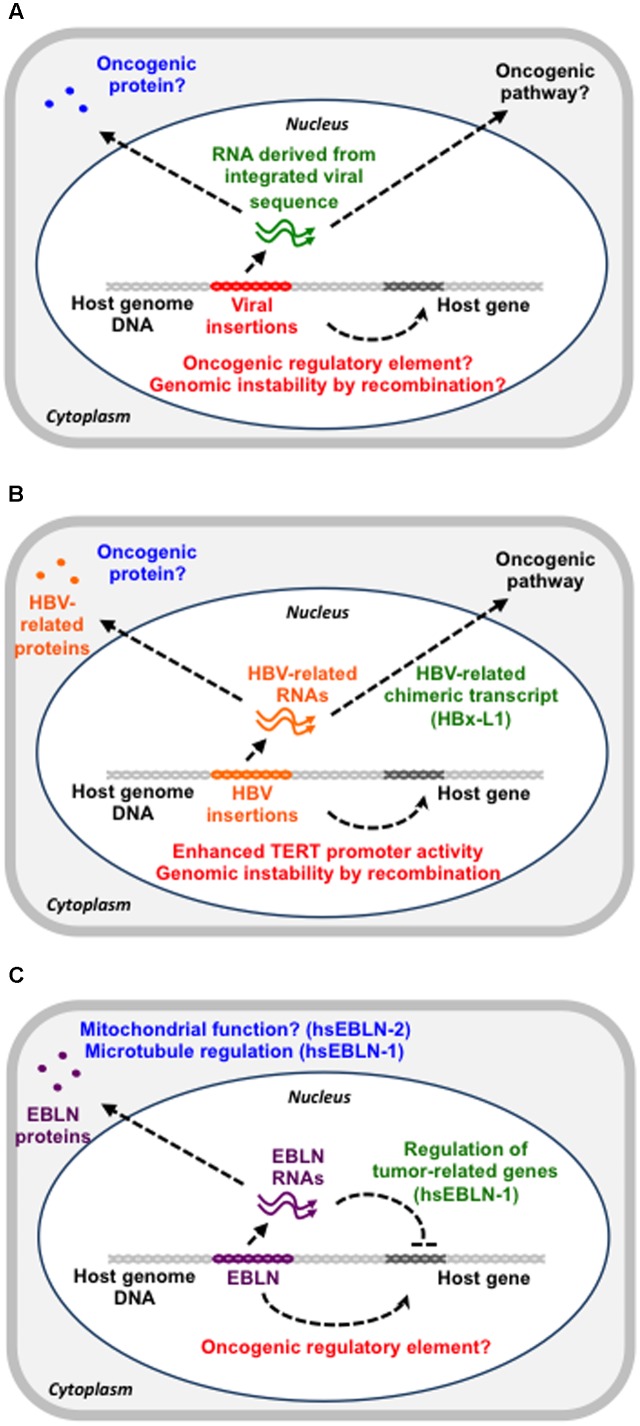
The possible contribution of viral sequences in the genome to cancer development. **(A)** Overview of possible functions of genomic viral sequences. Viral sequences in the genome could function as a gene regulatory DNA element (red), a functional RNA (green) or a protein (blue), all of which can contribute to cancer development. **(B)** HBV insertions in the genome. HBV insertions may enhance TERT promoter activity, have a recombinogenic effect or produce a viral-host chimeric RNA with an oncogenic potential. **(C)** EBLNs in the genome. The hsEBLN-2 protein may be involved in mitochondrial function, whereas the hsEBLN-1 protein may regulate microtubules. The hsEBLN-1 RNA has also been shown to regulate tumor-related genes.

Viral integration can have oncogenic potential via several mechanisms (**Figure 1A**). First, the inserted sequences in the vicinity of an oncogene may function as a promoter for the oncogene. Second, such events may inactivate tumor suppressor genes via insertional mutagenesis. Third, such integrated sequences may induce genomic instability via homologous recombination (Hino et al., 1991). Fourth, the integrated sequences may epigenetically regulate the host gene expression landscape, leading to cancer formation and spreading (Zhao et al., 2016). Fifth, such sequences may produce an oncogenic protein or non-coding RNA (Lau et al., 2014).

Regarding the relationship between viruses and cancers, many excellent reviews have been published about the links between endogenous retroviruses or tumor viruses and cancers (Suntsova et al., 2015; Gonzalez-Cao et al., 2016; Gramolelli and Ojala, 2017; McBride, 2017; Pancholi et al., 2017). On the other hand, few have dealt with the association between non-retroviral viral sequences in the genome and cancers. One of such studies has proposed that insertions of human papilloma virus contribute to cervical cancer formation through interrupting tumor suppressor or destabilizing chromosomes (Zhao et al., 2016). Here, I especially focus on the genomic sequences derived from two non-retroviral viruses, whose endogenization is already reported in animal genomes (Horie et al., 2010; Shen et al., 2016), and discuss the possibilities how these specific sequences could contribute to cancer formation. As DNA virus-related EVEs, EVEs derived from hepadnavirus and human herpesvirus 6 (HHV-6) are reported in animal genomes (Gravel et al., 2015; Shen et al., 2016). Because the link between HHV-6 insertions and cancer is not convincing at present, I will introduce current understanding regarding the roles of hepatitis B virus (HBV), a tumor-related hepadnavirus, insertions and hepatocellular carcinoma (HCC). Then, I will discuss the possible involvement of EVEs derived from ancient non-retroviral RNA virus sequences in cancers. Because endogenous bornavirus-like elements (EBLs) are the only non-retroviral RNA virus-derived EVEs found in the human genome thus far (Horie et al., 2010), I focus on the possible links between these elements and cancers although these links have not been demonstrated. EBLs are possibly generated in a retrotransposon-dependent manner. Therefore, I will finally propose the possible contribution of virus-retrotransposon interactions to cancers. This article aims to inspire future studies regarding the possible links described here, which may open a new avenue for understanding of the significance of viral insertions in the host genome.

## A Potential Link Between HCC and HBV Insertions in the Genome

Hepatocellular carcinoma accounts for 80% of liver cancer, whose major causative agents are two hepatitis viruses, HBV and hepatitis C virus (HCV) (Jemal et al., 2011; Forner et al., 2012; Tateishi and Omata, 2012). HBV is a DNA virus that belongs to the *Hepadnaviridae* family (Beck and Nassal, 2007; Nguyen et al., 2008), while HCV is an RNA virus and belongs to the *Flaviviridae* family (Hijikata et al., 1991; Grakoui et al., 1993; Aly et al., 2012). Both viruses can cause chronic infections, which may increase the chance of horizontal viral gene transfer to the host genome (Parkin, 2006; Aly et al., 2012). Consistent with this idea, EVEs derived from an ancient hepadnavirus and an ancestor HCV have been identified in animal genomes although they are not in the human genome. The budgerigar genome contains two EVEs with the full-length genome of the ancient budgerigar hepadnavirus (Shen et al., 2016). The rabbit and hare genomes have fragments homologous to HCV genes, which might suggest the possibility that cDNA from an HCV ancestor was integrated into the host genome (Silva et al., 2012). Although HCV replicates without a known DNA intermediate stage, it is still possible that the sequences of non-retroviral RNA viruses are integrated into the host genome via host retrotransposon machineries as evidenced by several studies (Geuking et al., 2009; Horie et al., 2010). HCV cDNA has been reportedly detected in patients infected with HCV (Zemer et al., 2008), further supporting this possibility. However, the contribution of integration events of the HCV sequences to oncogenesis remains unclear.

On the other hand, insertions of the HBV sequences seem to be closely linked to HCC development because the frequency of HBV insertions in cancer tissue is larger than that in cancer-adjacent tissues (Ding et al., 2012; Jiang et al., 2012). So far, several genes that are recurrently targeted by HBV insertions have been reported (Ding et al., 2012; Fujimoto et al., 2012). It has been proposed that HBV insertions occur during chronic hepatitis and that some of the cells with HBV insertions can acquire growth advantages and initiate tumorigenesis (Ding et al., 2012). A possible oncogenic contribution of HBV insertions is modification of gene expression via insertions into the genomic regulatory region, genomic instability induced by recombination between integrated HBV sequences or production of oncogenic cellular-HBV chimeric proteins or non-coding RNAs (**Figure 1B**). One of the first cases is the recurrent insertion into the telomerase reverse transcriptase (*TERT*) gene (Ferber et al., 2003). TERT expression is a limiting factor in telomerase activation and its upregulation is thought to be a critical step in tumorigenesis (Ferber et al., 2003). HBV insertions in the promoter region of the *TERT* gene enhance its expression, which might be related to HCC development (Ding et al., 2012; Sung et al., 2012). The second possibility is supported by the observation that fragments containing the HBV sequences increase the recombination events (Hino et al., 1991).

The chimeric gene, *HBx-L1*, is an example of the third possible mechanism described above (Lau et al., 2014). *HBx-L1* is a fusion gene of HBx, an HBV gene, and LINE-1, a host retrotransposon, produced by the HBV integration event, which is found in more than 20% of HBV-related HCC and correlates with a poor outcome (Lau et al., 2014). Knockdown of the HBx-L1 transcript reduces migratory and invasive properties of HBV-positive HCC cells. HBx-L1 overexpression confers a growth advantage and promotes cell migration and invasion via β-catenin/Wnt signaling, a major pathway in the oncogenesis of HBV-related HCC, regardless of its protein-coding potential (Lau et al., 2014). Thus, the HBx-L1 transcript is a chimeric long non-coding RNA (lncRNA) that promotes the HCC phenotype (Whittaker et al., 2010; Lau et al., 2014).

## A Potential Link Between Cancers and Endogenous Bornavirus-Like Elements

Endogenous bornavirus-like elements are the only non-retroviral RNA virus-derived EVEs found in the human genome, although DNA virus-derived EVEs are also found in the human genome (Gravel et al., 2015). The majority of such elements are EBLs from the bornavirus nucleoprotein (N) gene (EBLNs), which appear to have originated from the reverse-transcription and integration of ancient bornavirus N mRNA (Horie et al., 2010). Among 7 *Homo sapiens* EBLNs (hsEBLNs) in the human genome, hsEBLN-2 is most closely linked to cancer. Whole exome sequencing using two sibling pairs of non-smokers with lung adenocarcinoma reveals that a truncated mutation in hsEBLN-2 is only detected in affected siblings (Renieri et al., 2014). The authors concluded that this mutation in hsEBLN-2 might predispose an individual to lung adenocarcinoma (Renieri et al., 2014). The loss of 3p12-p14 is recurrently observed in uterine cervical cancer, suggesting a strong selection advantage for the gene loss (Lando et al., 2013). hsEBLN-2 is highly down-regulated in cases with this gene loss (Lando et al., 2013). Gene ontology analysis of the genes associated with the loss, including hsEBLN-2, shows enrichment of tumorigenic pathways, such as apoptosis, proliferation and stress responses, suggesting that hsEBLN-2 might be a tumor suppressor. hsEBLN-2 is homologous to the bornavirus N gene but also contains an additional TOM20 recognition motif (F_4_LKLY_8_) at the N-terminal. Furthermore, the hsEBLN-2 protein was shown to be expressed and to interact with several other host proteins (Ewing et al., 2007). Because mitochondrial dysfunction is found in cancers (Lleonart et al., 2017), hsEBLN-2 might play important roles in mitochondrial function and then act as a tumor suppressor (**Figure 1C**).

hsEBLN-1 retains a long open reading frame (ORF) that encodes 366 amino acids, which is comparable with the full-length BDV N protein (Horie et al., 2010). Despite the overall homology between hsEBLN-1 and BDV N proteins, their subcellular localizations are different, suggesting that hsEBLN-1 may have acquired new or additional functions during millions of years of residence within the human genome (Honda and Tomonaga, 2013; Fujino et al., 2014). Recently, two studies have revealed the involvement of hsEBLN-1 in tumorigenic pathways, such as cell cycle transit, cell genome stability and apoptosis (He et al., 2016; Myers et al., 2016) (**Figure 1C**). Both studies demonstrated that hsEBLN-1 silencing increases the proportion of cells in the G2/M phase. hsEBLN-1 knockdown cells exhibit microtubule and centrosomal splitting defects (Myers et al., 2016). Proteomic analysis of the purified hsEBLN-1 complex identified several binding partners for hsEBLN-1 (Myers et al., 2016). Among these, TPR (Translocated Promoter Region) is a nuclear protein that regulates mRNA transport and mitotic spindles. Because hsEBLN-1 silencing impairs the nuclear envelope localization of TPR, improper localization of TPR may abrogate TPR function to regulate microtubules and thereby induces abnormal cell cycle progression. Indeed, TPR has been implicated in cancer development (Snow and Paschal, 2014). In addition to this, three genes upregulated after hsEBLN-1 silencing, *RND3*, *OSMR*, and *CREB3L2*, are closely linked to glioma (He et al., 2016). This observation raises the possibility that hsEBLN-1 may be involved in the development of some kinds of cancers, although no hsEBLN-1 mutations have been identified in cancer thus far.

We have previously demonstrated that hsEBLN-1 can modulate the expression of its neighboring gene, *COMMD3* (Sofuku et al., 2015). When transcription from the hsEBLN-1 locus in the human genome was induced, expression of the *COMMD3* gene was downregulated. The effect of hsEBLN-1 RNA expression on the *COMMD3* locus was abrogated by treatment with siRNA against hsEBLN-1 RNA. These results suggest that hsEBLN-1 RNA may function as a lncRNA that scaffolds transcriptional and/or epigenetic repressors for the *COMMD3* gene and suppress its expression. Although we cannot exclude the possibility that hsEBLN-1 functions as a *cis*-regulatory DNA element or a protein acting on this locus *in trans*, our data using siRNA and cytoplasmic localization of the hsEBLN-1 protein strongly suggest a role for hsEBLN-1 as a lncRNA (Fujino et al., 2014; Sofuku et al., 2015). The *COMMD3* gene encodes a protein that can interact with and inhibit the NF-kB pathway (Burstein et al., 2005), which regulates type I interferons (IFNs), inflammatory cytokines, such as interleukin-1 (IL-1), IL-2, IL-6, IL-12, and tumor necrosis factor (TNF)-α and intercellular adhesion molecule 1 (ICAM-1). In addition, enhanced expression of the *COMMD3* gene was reported in a particular type of leukemia (Mulaw et al., 2012). EBLN insertion in the hsEBLN-1 locus may downregulate the expression of the *COMMD3* gene and thereby potentiate the NF-kB pathway (Honda and Tomonaga, 2016). Cancer cells are known to induce IFNs, which mediate anti-tumor effects on particular types of tumors, such as renal cell carcinoma, and are therefore used in clinical anti-cancer therapy (Müller et al., 2017; Wu et al., 2017). Taken together, hsEBLN-1 may exert anti-tumor effects via the COMMD3-NF-kB-IFN pathway. Further studies are required to understand the contribution of EBLNs to immune modulation during oncogenesis.

## Possible Involvement of Retrotransposon-Virus Interactions in Carcinogenesis

As described above, non-retroviral RNA virus-related sequences in the genome are possibly generated by a retrotransposon machinery (Horie et al., 2010; Shimizu et al., 2014). In other words, retrotransposons are a major driving force for generating such EVEs. Therefore, it is important to understand the interactions between retrotransposons and viruses. Among retrotransposons, L1s constitute approximately 17% of the human genome (Lander et al., 2001). Most L1s are 5′ truncated and therefore defective in retrotransposition, whereas 80–100 copies are still retrotransposition-competent and utilize a “copy-and-paste” mechanism to retrotranspose to new genomic loci (Beck et al., 2010; Brouha et al., 2003). L1 is also responsible for the production of non-retroviral RNA virus elements in the host genome as described. Thus, dysregulation of L1s is considered a major source of endogenous insertional mutagenesis in humans (Levin and Moran, 2011; Burns and Boeke, 2012). Indeed, L1 retrotransposition occurs not only in germ line cells and pluripotent stem cells (van den Hurk et al., 2007; Beck et al., 2011; Levin and Moran, 2011; Klawitter et al., 2016) but also in cancer cells (Iskow et al., 2010; Goodier, 2014). Furthermore, although it is unclear whether L1s are activated in normal cells before clonal expansion or in cancer cells during the later stages of carcinogenesis (Goodier, 2014), many epidemiological studies suggest a linkage between dysregulated L1 expression and cancers (Shukla et al., 2013; Rodić et al., 2014; Harada et al., 2015). Once L1 or L1-mediated viral insertions occur around oncogenes or tumor suppressor genes, some of these insertions may confer survival and/or proliferative advantages to the cells, thereby enhancing the various steps of carcinogenesis. Consistent with this idea, transposon-based insertional mutagenesis has been shown to induce virtually any kind of cancer in mice (Dupuy et al., 2005, 2009; Rad et al., 2010). Furthermore, several tumor viruses are reported to activate transcription of retrotransposons, such as endogenous retroviruses and short interspersed nuclear elements (SINEs). For example, Marek’s disease virus, an avian tumor virus, is reported to induce expression of an endogenous retrovirus (Hu et al., 2017), and murine gammaherpesvirus 68, another tumor virus, also activates transcription of SINEs (Tucker and Glaunsinger, 2017). These observations may emphasize the significance of retrotransposon activation in tumor virus-related carcinogenesis.

## Conclusion and Perspective

This article has presented a current view of the possible contributions of hepadnavirus and bornavirus insertions in the genome to cancer formation. The presented lines of evidence suggest potential links between these viral sequences and cancers. However, current knowledge in this field is still poor, and there are many questions to be addressed. Although several genes recurrently targeted by HBV insertions have been identified, the precise role of most of them in tumorigenesis remains unclear. Among the HBV integration sties identified so far, only a limited number of cellular-HBV chimeric proteins/transcripts have demonstrated the oncogenic potential. Further accumulation of examples of recurrent HBV insertion sites in the host genome or recurrent chimeric transcripts specific to hepatitis virus-related HCC will be promising to understand the contribution of HBV insertions to HCC etiology. Regarding links between EBLs and cancers, the information is more limited. Epidemiological studies on the links between EBL mutations and cancers are clearly required. Furthermore, the causal relationship between such EBL mutations and cancers should be demonstrated in future.

Although a definitive role for tumor viruses in retrotransposon activation has not been established thus far, investigating a possible link between L1 activation and tumor viruses, especially HBV, would be of considerable interest because L1 hypomethylation or some L1 chimeric transcripts are associated with poor prognosis in HCC (Honda, 2016). Hypomethylation of the L1 loci may upregulate L1 expression, potentially removing an obstacle to L1 transposition in liver cells. Once L1s are activated, any potential disruption of tumor suppressor genes induced by L1 retrotransposition could contribute to the development of HCC. Indeed, L1 has been shown to be a crucial source of mutations that can reduce the tumor-suppressive capacity of somatic cells (Shukla et al., 2013).

Future studies regarding the above links may open a new avenue for the development of novel therapeutics, such as epigenetic modification of viral sequences in the genome, for tumor virus-related cancers. Also, such studies will provide novel insights into the biological roles of EVEs in the cells.

## Author Contributions

TH wrote the manuscript, confirms being the sole contributor of this work and approved it for publication.

## Conflict of Interest Statement

The author declares that the research was conducted in the absence of any commercial or financial relationships that could be construed as a potential conflict of interest.
